# Association between duration of hearing-aid use and frailty in community-dwelling older adults with hearing loss in Japan: a cross-sectional study

**DOI:** 10.1186/s12877-026-07434-6

**Published:** 2026-04-07

**Authors:** Madoka Tatsukawa, Ayumi Kono

**Affiliations:** 1 Department of Community-based Integrated Care Science, School of Nursing, Osaka Metropolitan University, Osaka, Japan; 2 Department of Nursing, Graduate School of Medicine, Ehime University, Ehime, Japan

**Keywords:** Frailty, Hearing aids, Hearing loss, Older adults

## Abstract

**Background:**

Frailty is prevalent among older adults with hearing loss. —a condition potentially triggering social isolation, communication difficulties, and reduced participation in physical and social activities, which in turn may accelerate the development of frailty. Hearing aids help mitigate these consequences by improving communication and social engagement. However, the relationship between duration of hearing-aid use and frailty is unclear. This study examined the above-mentioned relationship in older adults with hearing loss.

**Methods:**

A cross-sectional survey was conducted between June and August 2024 at 12 hearing- aid specialty stores in Ehime Prefecture, Japan. The participants were aged ≥ 65 years and reported hearing loss, defined as a PTA of ≥ 25 dB. Multivariate logistic regression was used to examine the association between duration of hearing-aid use (< 8, ≥ 8 to < 12, and ≥ 12 h per day) and frailty, which was treated as a binary variable (1 = frail [FSI score ≥ 3], 0 = non-frail [FSI score 0–2]), adjusting for age, sex, household, education level, economic status, employment, aided ear, history of ear disease, hearing-aid use method and frequency, period of hearing-aid use, PTA, and fitting evaluation based on sound-field thresholds.

**Results:**

A total of332 participants (female: 56.3%; mean age [standard deviation]: 81.30[6.93]) were categorized into three groups based on duration of hearing-aid use: <8 h (*n* = 94), ≥ 8 h to < 12 h (*n* = 80), and ≥ 12 h (*n* = 158). Compared to those using hearing aids for ≥ 8 h to < 12 h, those in the < 8 h group had significantly higher odds of frailty (odds ratio: 3.32; 95% confidence interval: 1.09–12.17; *p* = .048).

**Conclusions:**

Shorter daily hearing-aid use (< 8 h) was associated with greater frailty among older adults with hearing loss. Given this study’s cross-sectional design, causal inferences cannot be made. Longitudinal studies are warranted to determine whether appropriate hearing- aid use can help prevent frailty in this population.

**Supplementary Information:**

The online version contains supplementary material available at 10.1186/s12877-026-07434-6.

## Background

The global population of people with hearing loss stood at 466 million in 2018 and is projected to reach 630 million by 2030 and 900 million by 2050 [1]. Hearing loss among older adults is associated with depression [2], falls [3], and cognitive decline [4], which ultimately contribute to the progression of frailty [5].

Frailty is characterized by increased vulnerability to stressors due to a decline in physiological reserve capacity [6]. It is associated with an increased risk of falls, longer hospitalization, and mortality [7–9], leading to a shorter healthy life expectancy. Importantly, frailty is a dynamic and potentially reversible condition if appropriate interventions are implemented [10–12]. Longitudinal studies have indicated that older adults with hearing loss are more likely to transition from a non-frail state to a pre-frail or frail state over time [13]. It is held that communication difficulties resulting from hearing loss may increase cognitive burden [14] and social isolation [15], thereby accelerating frailty progression [16]. Since hearing loss is a potentially modifiable risk factor, interventions to mitigate its impact may be critical for preventing frailty.

One effective way to compensate for hearing loss is the use of hearing aids. Several recent studies have examined the health-related effects of hearing-aid use in older adults, with certain works finding that hearing-aid use was associated with better health outcomes and improved quality of life, as well as a potential attenuation of cognitive decline [17–19]. Regular hearing-aid use improves speech perception over time, leading to better speech recognition [20], which in turn alleviates the stress associated with hearing difficulties and promotes smoother communication. Improved communication and reduced stress can enhance the quality of life of older adults with hearing loss and may help prevent frailty. These benefits emphasize the importance of the continuous and consistent use of hearing aids in managing hearing loss. However, previous studies [16, 21] concerning older adults with hearing loss have primarily focused on the presence or absence of hearing-aid use without investigating the association between duration of hearing-aid use and frailty. We hypothesize that the consistent daily use of hearing aids improves speech perception, facilitates communication, reduces cognitive load, and ultimately helps prevent frailty.

This study aims to investigate the association between duration of hearing-aid use and frailty in community-dwelling older adults with hearing loss in Japan.

## Methods

### Study setting and participants

This cross-sectional study involved a questionnaire survey targeting older adults with hearing loss who visited specialty hearing-aid stores. Participants were recruited from 12 hearing-aid specialty stores in the Ehime Prefecture, Japan, between June 1 and August 31, 2024, and met the following eligibility criteria: age ≥ 65 years (the typical retirement age in Japan), and a pure-tone average (PTA) of ≥ 25 dB in at least one ear. Individuals who could not complete the questionnaire independently were excluded. The study did not restrict participants based on their hearing-aid use history; both first-time users and those with prior experience were welcomed. This reflects the real-world diversity of hearing-aid users visiting specialty stores. In Japan, hearing aids can be purchased not only at specialty stores but also at optical stores or online. According to a survey by the Japan Hearing Instrument Manufacturers Association, approximately 50% of hearing-aid purchasers obtain their devices from specialty stores [22]. Although the overall hearing-aid adoption rate in Japan is low [22], conducting our study at specialty stores, which account for half of all purchases, allows for a certain degree of generalizability. The study area has a higher proportion of older adults compared to the national capital region, but is not particularly affluent, which reduces the likelihood of selection bias related to socioeconomic status. Furthermore, public financial assistance for hearing aids purchase in Japan is generally limited to individuals with severe hearing loss or worse [23]. Recent years have seen, some municipalities independently introduce subsidy programs for older adults with mild to moderate hearing loss, but the amount and availability of support vary by region [24].

The questionnaire was distributed to 557 individuals, with 417 responding. After excluding 20 participants who were aged under 65 and did not meet the eligibility criteria, one duplicate respondent, and one participant who withdrew consent, 395 participants were included in the initial analysis. Among them, 332 participants had complete data for the dependent variable, independent variable, and covariates, and were therefore included in the final analysis.

### Data collection

Data were collected from 12 hearing-aid specialty stores in Ehime Prefecture that agreed to participate in the study. Store staff at these locations distributed a set of materials to eligible participants, including a study information sheet, consent form, questionnaire, and a pre-addressed return envelope. After reading the information sheet and agreeing to participate, those who consented signed the consent form, completed the questionnaire, and returned both documents to the researcher by mail or by handing them to store staff. Questionnaires collected at the stores were subsequently retrieved by the researcher through scheduled visits. The questionnaire was primarily self-administered. However, when participants requested assistance and provided consent, researchers or store staff supported them in an interview format. This support was limited to facilitating the mechanics of questionnaire completion, such as reading items aloud or recording verbal responses. Prior to data collection, the researcher explained the study’s purpose and procedures to store managers, who then conveyed the information to their staff. Staff involvement was limited to distributing study materials, assisting with the consent process, and providing questionnaire support upon participant request. Importantly, staff were not involved in interpreting or scoring responses.

The questionnaire assessed duration of hearing-aid use, frailty, demographic characteristics, aided ear, history of ear disease, method and frequency of hearing-aid use, and the age at which they first began using hearing aids. With participant consent, audiometric data were obtained from the hearing-aid stores to evaluate hearing ability and hearing-aid fitting. These data included pure-tone audiometry thresholds obtained at the time of hearing-aid purchase and the sound-field threshold measurements conducted closest to the date of questionnaire distribution.

### Independent variables

#### Duration of hearing-aid use

To measure the duration of hearing-aid use, we asked the participants, “Over the past month, how many hours per day have you typically worn your hearing aids?” If they removed and reinserted their hearing aids during the day, they were instructed to report the cumulative duration of use. The duration of hearing-aid use was categorized into three groups― < 8 hours, ≥ 8 to < 12 hours, and ≥ 12 hours per day,―with ≥ 8 to < 12 hours set as the reference group. Although no standardized cutoff values for duration of hearing-aid use have been established in the literature, in this study the classification was determined based on previous research [19–20, 22, 25–26] and typical daily activity patterns, with ≥ 8 hours to < 12 hours defined as the reference category.

#### Outcome

The outcome was frailty, which was assessed using the Frailty Screening Index (FSI) developed by Yamada et al. [27]. The FSI has been validated for reliability and predictive validity in older Japanese adults [27]. The FSI is based on the phenotypic model from Fried et al. [28], and comprises five yes/no questions: “Have you lost 2–3 kg in the past 6 months?” (Yes = 1), “Do you think you walk slower than before?” (Yes = 1), “Do you go for a walk for your health at least once a week?” (No = 1), “Can you recall what happened 5 minutes ago?” (No = 1), and “In the past 2 weeks, have you felt tired without a reason?” (Yes = 1). Each item was scored as 0 or 1, with the total score ranging from 0 to 5. A score of 3 or more was defined as frail, 1 or 2 as pre-frail, and 0 as robust.

### Covariates

#### Demographic characteristics

Data on the following demographic characteristics were collected: sex, age, educational level, economic status, employment status, and household status. Education level was categorized as ≤ 12 years and ≥ 13 years, corresponding to high school education or less, and education beyond high school (e.g., vocational school, junior college, or university), respectively, based on the structure of the Japanese education system. Economic status was assessed based on the degree of financial comfort in affording hobbies and leisure, with responses ranging from 1 (“not at all”) to 5 (“very much”). Participants were then classified as financially constrained (scores of 1–3) or financially comfortable (scores of 4–5). This subjective measure was developed specifically for the current study. Because older adults may be reluctant to disclose exact income, perceived financial comfort was considered a practical indicator of daily living conditions. Households were classified as living alone or not living alone.

#### Basic information regarding hearing-aid use

##### Aided ear

To determine whether participants used hearing aids bilaterally or unilaterally, the questionnaire included an item asking which ear(s) they typically wore their hearing aid(s) in.

##### History of ear disease

To assess participants’ history of ear disease, they were asked to select one of the following options: “sudden sensorineural hearing loss”, “otitis media”, “other ear conditions”, or “no history”. The participants were then classified into two groups: those who selected sudden sensorineural hearing loss, otitis media, or other ear conditions were considered to have a history of ear disease. The variable was then dichotomized accordingly.

##### Hearing-aid use method

Hearing-aid use method was assessed using the following question: “Do you wear your hearing-aid all day or remove it intermittently?” Those stating that they wore it all day were classified as regular users, whereas those stating that they put it on and remove it intermittently were classified as intermittent users.

##### Frequency

Frequency of hearing-aid use was assessed using the following question: “How often do you wear your hearing-aid?” Responses were categorized into specific options: “Every day,” “2–3 times per week,” “Once per week,” “2–3 times per month,” and “Once per month.” The participants were then classified into two groups: those who wore hearing aids every day and those who wore them less frequently. This covariate was included based on prior research suggesting that regular hearing-aid use may improve communication and hearing ability and potentially prevent cognitive decline―while also enhancing quality of life―in older adults [19–20, 26].

##### Period of hearing-aid use

Period of hearing-aid use was calculated by asking participants the age at which they first began using hearing aids and subtracting this from their current age. For the analysis, period of hearing-aid use was dichotomized into < 1 year and ≥ 1 year, because adjustment to hearing-aid use generally requires time, and after approximately one year, users are more likely to have adapted to the device and to maintain stable patterns of use.

#### Pure tone average (PTA)

The PTA was determined using pure-tone audiometry data at the time of hearing-aid purchase, which were provided by hearing-aid specialty stores. The PTA was calculated using the Four-Frequency PTA Method B, which averages the thresholds at 500, 1,000, 2,000, and 4,000 Hz. According to the Japan Audiological Society, hearing loss is classified as mild for thresholds of 25 to < 40 dB, moderate for 40 to < 70 dB, severe for 70 to < 90 dB, and profound for 90 dB or higher [29]. The PTA was calculated using data from the better-hearing or aided ear.

#### Hearing-aid fitting evaluation

The hearing-aid fitting was evaluated using sound-field threshold measurements. This evaluation aimed to determine whether the hearing-aid was appropriately fitted based on the hearing thresholds measured in a sound field. A hearing-aid was considered well-fitted if one or both of the following criteria were met: the aided sound-field threshold at 1,000 Hz was ≤ 35 dB or functional gain was confirmed by comparing unaided and aided sound-field thresholds after fitting, with improvements observed at 500 Hz, 1,000 Hz, and 2,000 Hz.

### Statistical analysis

Descriptive statistics were used to summarize participants’ demographic characteristics. Continuous variables were presented as means and standard deviations, while categorical variables were reported as frequencies and percentages.

To investigate the association between duration of hearing-aid use and frailty, univariate and multivariate logistic regression analyses were performed. The dependent variables were frailty status. Frailty was dichotomized by combining “robust” and “pre-frail” as 0 and coding “frail” as 1. Logistic regression models were applied to this binary outcome.

In the multivariate analyses, adjustments were made for potential confounders, including age, sex, educational level, economic status, household composition, employment status, the aided ear, history of ear disease, method and frequency of hearing aid use, period of hearing-aid use, PTA, and fitting evaluation based on the sound-field threshold. The covariates included in the regression models were selected based on prior research [30–32] and theoretical considerations of factors likely to influence hearing-aid use duration. Although formal pre-assessment of their confounding potential was not conducted, these variables were incorporated to account for possible confounding effects. Multicollinearity among independent variables was assessed using variance inflation factors (VIFs). For categorical variables, generalized VIFs (GVIFs) were calculated and adjusted based on degrees of freedom using the formula GVIF^(1/(2×Df)). All adjusted VIF values were below 5, indicating no concerning multicollinearity in the model. All analyses were conducted using R software (version 4.4.0), and the level of statistical significance was set at 5%.

### Sensitivity analysis

To assess the robustness of the main findings, a sensitivity analysis was conducted. Specifically, each of the five components of frailty— weight loss, slow walking speed, low physical activity, memory impairment, and exhaustion—was treated as an individual outcome. The sensitivity analysis was conducted using logistic regression models, with explanatory variables and covariates identical to those used in the main analysis.

## Results

### Demographic characteristics

Table 1 shows the demographic characteristics of the study participants. The mean age of the 332 participants was 81.30 (SD 6.93). Of these, 187 (56.3%) were female, 242 (72.9%) lived with others, 246 (74.1%) had ≤ 12 years of education, 227 (68.4%) reported being financially comfortable, and 54 (16.3%) were employed. Regarding duration of hearing-aid use, 94 participants reported using a hearing-aid for < 8 h, 80 for ≥ 8 to < 12 h, and 158 for ≥ 12 h (Table 1).

**Table 1:** Characteristics and frailty of study participants(*N* = 332)

Variable	Total	Duration of hearing-aid use		
	(*N* = 332)	< 8 h(*N* = 94)	8 to < 12 h(*N* = 80)	≧ 12 h (*N* = 158)
Age (mean(SD)	81.30 (6.93)	81.33 (6.47)	81.34(7.01)	81.25 (7.18)
Sex				
female, n (%)	187 (56.3)	56 (59.6)	44 (55.0)	87 (55.1)
Household				
living with others, n (%)	242 (72.9)	64(68.1)	57(71.2)	121(76.6)
Education level				
12 years or less, n (%)	246 (74.1)	71 (75.5)	59 (73.8)	116 (73.4)
Economic status				
financially comfortable, n (%)	227 (68.4)	65 (69.1)	61 (76.2)	101 (63.9)
Employment				
employed, n (%)	54 (16.3)	14 (14.9)	15 (18.8)	25 (15.8)
Frailty ^a^				
robust, n (%)	60 (18.1)	17 (18.1)	16 (20.0)	27 (17.1)
pre-frail, n (%)	195 (58.7)	51 (54.3)	49 (61.3)	95 (60.1)
frail, n (%)	77 (23.2)	26 (27.7)	15 (18.8)	36 (22.8)

^a^ Frailty was assessed via the Frailty Screening Index (the FSI). A total score of 3 or more indicates frailty, a score of 1 or 2 indicates pre-frailty, and a score of 0 indicates robustness

### Basic information on hearing-aid use

Table 2 presents the information on hearing-aid use, including duration, among the study participants. The average duration of hearing-aid use was 10.33 h (SD 4.51). Of the participants, 197 (59.3%) used hearing aids bilaterally. A total of 210 participants (63.3%) were regular users, and 289 (87.0%) wore hearing aids daily. Among them, 40 participants (12.0%) had been using hearing aids for < 1 year. A history of ear disease was reported by 142 participants (42.8%).The PTA at the time of hearing-aid purchase was 53.90 dB (SD 10.82). In the evaluation of hearing-aid fitting based on sound-field thresholds, 245 participants (73.8%) were classified as well-fitted (Table 2).

**Table 2:** Duration of hearing-aid use and basic information of hearing-aid use among study participants　(*N* = 332 )

Variable	Total	Duration of hearing-aid use		
	(*N* = 332)	< 8 h(*N* = 94)	8 to < 12 h(*N* = 80)	≧ 12 h (*N* = 158)
Duration of hearing-aid use, hours, (mean (SD)	10.33 (4.51)	4.37 (1.75)	9.64 (1.18)	14.23 (1.88)
Aided ear				
bilateral ears, n (%)	197(59.3)	61(64.9)	50(62.5)	86(54.4)
Hearing-aid use method				
regular user, n (%)	210 (63.3)	3 (3.2)	56 (70.0)	151 (95.6)
History of ear disease				
Positive history of desease, n(%)	142(42.8)	38(40.4)	29(36.2)	75(47.5)
Frequency of hearing-aid use				
every day, n(%)	289 (87.0)	62 (66.0)	71 (88.8)	156 (98.7)
2-3 times per week	36(10.8)	25(26.6)	9(11.2)	2(1.3)
Once per week	4(1.2)	4(4.3)	0(0.0)	0(0.0)
2–3 times per month	1(0.3)	1(1.1)	0(0.0)	0(0.0)
Once per month	2(0.6)	2(2.1)	0(0.0)	0(0.0)
Period of hearing aid use				
< 1 year	40(12.0)	15(16.0)	12(15.0)	13(8.2)
Pure tone average (PTA), Decibels Hearing Level(dBHL)^a^ , (mean (SD)	53.90 (10.82)	51.88 (10.63)	53.34 (8.96)	55.38 (11.60)
Hearing-aid fitting evaluation				
Sound field threshold ^b^				
well-fitted, n (%)	245 (73.8)	73 (77.7)	60 (75.0)	112 (70.9)

^a^ The PTA was calculated using data from the better-hearing ear or the aided ear

^b^A hearing-aid was considered well-fitted if one or both of the following criteria were met: 1) The aided sound field threshold at 1000 Hz was ≤ 35 dB; or2) Functional gain was confirmed by comparing unaided and aided sound field thresholds after fitting, with improvements observed at 500 Hz, 1000 Hz, and 2000 Hz

### Association between duration of hearing-aid use and frailty

Among the 332 participants, frailty status data indicated that 60 participants (18.1%) were classified as robust, 195 (58.7%) as pre-frail, and 77 (23.2%) as frail. In the univariate analysis, the duration of hearing-aid use for < 8 h (odds ratio [OR]: 1.40, 95% confidence interval [CI]: 0.80–2.41 *p*=.227), ≥ 8 to < 12 h (OR: 0.71, 95% CI: 0.37–1.30 *p*=.281), and ≥ 12 h (OR: 0.96, 95% CI: 0.57–1.59 *p*=.867) was not significantly associated with frailty (Table 3). In the adjusted analysis, the duration of hearing-aid use for < 8 h was significantly associated with frailty compared to a duration of ≥ 8 to < 12 h (OR: 3.32, 95% CI: 1.09–12.17 *p*=.048). In contrast, the duration of hearing-aid use for ≥ 12 h was not significantly associated with frailty (OR: 1.03, 95% CI: 0.50–2.20 *p*=.929) (Table 3).

**Table 3:** Association between duration of hearing-aid use and frailty according to logistic regression analysis(*N* = 332)

Independent variables	Unadjusted ^a^	Adjusted ^b^
	OR (95% CI)	OR (95% CI)
Duration of hearing aid use		
8 to < 12 h (*n* = 80)	0.71 (0.37–1.30)	Ref
< 8 h (*n* = 94)	1.40 (0.80–2.41)	3.32* (1.09–12.17)
≧ 12 h (*n* = 158)	0.96 (0.57–1.59)	1.03 (0.50–2.20)

The dependent variables were robustness, pre-frailty (reference), and frailty

*OR* odds ratio, *CI* confidence interval

*p<.05

^a^ Unadjusted estimates are derived from univariate logistic regression analyses

^b^ Multivariate logistic regression analysis was performed adjusted for age, sex, education level, economic status, household, employment, aided ear,　history of ear disease, hearing-aid use method, frequency of hearing-aid use, period of hearing-aid use, pure-tone average (PTA), and fitting evaluation based on sound field threshold

### Sensitivity analysis result

As regards the sensitivity analysis, participants who used hearing aids for < 8 h showed a statistically significant association with exhaustion compared to those who used them for ≥ 8 to < 12 h (odds ratio: 3.63, 95% confidence interval: 1.44–10.08, *p*=.01). No statistically significant differences were observed for the other frailty components—weight loss, slow walking speed, low physical activity, and memory impairment(eTable1).

## Discussion

In this study, we examined the association between duration of hearing-aid use and frailty. We found that individuals who used hearing aids for < 8 h had a significantly higher risk of frailty compared to those who used them for ≥ 8 h to < 12 h.

Although the mechanisms linking hearing loss and frailty remain unclear, hearing loss can cause difficulties during communication and impose significant cognitive demands on sound processing [14]. Additionally, hearing loss is independently associated with social isolation [15], cognitive decline [4], reduced physical activity [33], falls [3], and difficulties in performing the activities of daily living [34], all of which may lead to frailty. One possible explanation for the association between using hearing aids for < 8 h and frailty is that limited use may not provide sufficient auditory compensation, thereby maintaining cognitive load and social isolation. Sensitivity analysis revealed a statistically significant association between duration of hearing-aid use for < 8 h and exhaustion, a key component of frailty. This finding suggests that insufficient auditory input may lead to sustained cognitive and psychological burden [14, 35], which can manifest as unexplained exhaustion. Although no significant associations were observed with other components of frailty—namely, weight loss, slow walking speed, low physical activity, and memory impairment—the direction of the odds ratios was consistent, indicating a tendency toward increased frailty-related symptoms among individuals with shorter hearing-aid use. These results underscore the importance of adequate auditory compensation in the prevention of frailty.

Among participants who used hearing aids for ≥ 12 h, no significant association with frailty was observed. However, this result should be interpreted with caution, considering this group’s background characteristics.

Compared to other groups, participants in the ≥ 12 h group tended to have poorer hearing thresholds and longer periods of hearing-aid use, alongside being less likely to report financial comfort. These findings suggest that prolonged hearing-aid use may reflect more severe hearing loss and greater daily challenges. In particular, limited financial resources may restrict access to regular device adjustments or higher-quality hearing aids, potentially resulting in suboptimal fitting and reduced auditory compensation. Resultantly, even with long hours of use, the expected benefits—such as improved communication and reduced cognitive load—may not be fully realized, and the pathway to frailty prevention may remain ineffective. These findings highlight the importance of considering not only the duration of hearing-aid use, but also the quality of use and the broader socioeconomic context.

Our findings suggest a potential association between duration of hearing-aid use and frailty. However, the wide CIs indicate a lack of precision in the effect estimation, which may be attributable to the relatively small sample sizes in the groups using hearing aids for < 8 h and for ≥ 8 to < 12 h compared with the group using them for ≥ 12 h: this may also be attributable to the imbalance in hearing-aid use frequency (daily vs. non-daily) and period of hearing-aid use (< 1 year vs. ≥ 1 year), which may have limited this study’s statistical power. Future studies with larger sample sizes are needed to improve the effect estimation accuracy and further explore the association between duration of hearing-aid use and frailty.

Although previous studies have suggested that hearing-aid use may contribute to the prevention of frailty [16, 21], they did not focus on duration of hearing-aid use. This study provides new insights by demonstrating that wearing hearing aids for < 8 h per day may be associated with higher odds of frailty, compared to those who wear them for ≥ 8 to < 12 h. These findings suggest that appropriate use of hearing aids may be beneficial, although causality cannot be inferred from this cross-sectional design. Appropriate fitting and guidance on proper use may be important for increasing the duration of hearing-aid use [36]. Therefore, the implementation of follow-up programs to encourage the continuous use of hearing aids is necessary to prevent frailty in older adults.

### Limitations

This study has certain limitations. First, due to its cross-sectional nature, it was not possible to establish a temporal order in the observed associations (whether duration of hearing-aid use preceded frailty or vice versa). Further research is needed to determine whether insufficient hearing-aid use is a risk factor for subsequent onset of frailty. Second, as this survey was a self-administered questionnaire, duration of hearing-aid use and frailty need to be objectively assessed.

Third, this study did not include data from speech-in-noise (SIN) testing, which is considered to better reflect hearing ability in daily life [37]. Since we live in environments with various background sounds, SIN testing may be useful for evaluating associations between hearing-aid use, frailty, and functional status. Incorporating SIN testing in future studies would allow for a more detailed assessment of the relationship between duration of hearing-aid use and frailty in everyday life. Fourth, we conducted the analysis using complete cases only. Although the proportion of missing data was below 20%, this approach may have introduced selection bias by excluding participants with incomplete data. Finally, this study was conducted in Japan, where cultural norms regarding hearing-aid use may differ from those in other countries. For example, there is a social stigma associated with hearing-aid use in Japan [22, 38], and public financial assistance for hearing-aid purchase is generally limited to individuals with severe hearing loss or worse [23]. Recent years have seen some municipalities independently introduce subsidy programs for older adults with mild to moderate hearing loss, but the amount of support varies by region [24]. These cultural and systemic factors may influence hearing-aid usage patterns and frailty risk. Therefore, caution is warranted when generalizing these findings internationally.

At the same time, unlike previous studies, which compared hearing-aid users and non-users, our study exclusively focused on hearing-aid users. This design reduces the potential selection bias stemming from differences between users and non-users, and provides a clearer understanding of the variation in frailty risk among individuals who have adopted hearing aids.

## Conclusion

This cross-sectional study suggests that the duration of hearing-aid use for < 8 h per day is associated with frailty in older adults with hearing loss. These findings provide a rationale for further longitudinal studies to clarify the long-term effects of the duration of hearing-aid use on frailty, and to explore whether optimizing hearing aid use could contribute to reducing frailty risk.

## Supplementary Information

Additional file 1: Flow of participants in the study. The diagram outlines the recruitment process, the number of participants excluded for reasons such as age, duplicate responses, or withdrawal of consent, and the final number of participants with complete data used for the statistical analysis.

Additional file 2: Association between duration of hearing-aid use and frailty components: a sensitivity analysis. This table presents the results of the sensitivity analysis, showing the robustness of the association between duration of hearing-aid use and frailty.

## Data Availability

The datasets used and/or analyzed during the current study are available from the corresponding author on reasonable request.

## Abbreviations

FSI: Frailty Screening Index
PTA: Pure-tone average
