# Supplementary figures and images for: Association between duration of hearing-aid use and frailty in community-dwelling older adults with hearing loss in Japan: a cross-sectional study
Source: BMC Geriatr. 2026 Apr 7;26:696. doi: 10.1186/s12877-026-07434-6 (PMC13191882; doi:10.1186/s12877-026-07434-6)

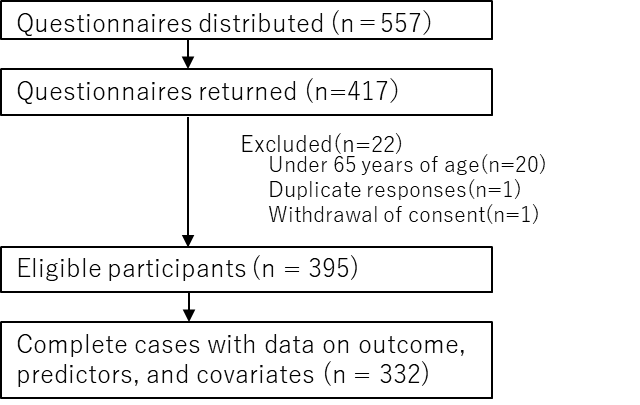


Figure1　Flow of participants in the study

Supplement: Supplementary file 1 — Additional file 1: Flow of participants in the study. The diagram outlines the recruitment process, the number of participants excluded for reasons such as age, duplicate responses, or withdrawal of consent, and the final number of participants with complete data used for the statistical analysis. [file 12877_2026_7434_MOESM1_ESM.docx]
