# Supplementary material for: Association between duration of hearing-aid use and frailty in community-dwelling older adults with hearing loss in Japan: a cross-sectional study
Source: BMC Geriatr. 2026 Apr 7;26:696. doi: 10.1186/s12877-026-07434-6 (PMC13191882; doi:10.1186/s12877-026-07434-6)
Supplement: Supplementary file 2 — Additional file 2: Association between duration of hearing-aid use and frailty components: a sensitivity analysis. This table presents the results of the sensitivity analysis, showing the robustness of the association between duration of hearing-aid use and frailty. [file 12877_2026_7434_MOESM2_ESM.docx]

etable2 Association between hearing-aid use duration and each component of frailty: sensitivity analysis(N=332)

|  |  | weight loss | | |  | slow walking speed | | |  | low physical activity | | |
| --- | --- | --- | --- | --- | --- | --- | --- | --- | --- | --- | --- | --- |
| Independent variables |  | Unadjusted ^a^ |  | Adjusted ^b^ |  | Unadjusted ^a^ |  | Adjusted ^b^ |  | Unadjusted ^a^ |  | Adjusted ^b^ |
|  |  | OR  (95% CI) |  | OR  (95% CI) |  | OR  (95% CI) |  | OR  (95% CI) |  | OR  (95% CI) |  | OR  (95% CI) |
| Duration of hearing-aid use |  |  |  |  |  |  |  |  |  |  |  |  |
| ≥8 to < 12 hours (n=80) |  | 0.96 (0.44-1.93) |  | Ref |  | 0.71 (0.42-1.20) |  | Ref |  | 1.20 (0.72-2.00) |  | Ref |
| < 8 hours (n=94) |  | 1.09 (0.54-2.10) |  | 1.28 (0.39-4.83) |  | 1.00 (0.61-1.67) |  | 1.10 (0.45-2.64) |  | 0.83 (0.51-1.36) |  | 1.21 (0.50-3.03) |
| ≥12 hours (n=158) |  | 0.96 (0.52-1.79) |  | 0.86 (0.36-2.11) |  | 1.29 (0.82-2.05) |  | 1.68 (0.88-3.22) |  | 1.01  (0.65-1.57) |  | 0.72 (0.39-1.32) |

|  |  | memory impairment | | |  | exhaustion | | |
| --- | --- | --- | --- | --- | --- | --- | --- | --- |
| Independent variables |  | Unadjusted ^a^ |  | Adjusted ^b^ |  | Unadjusted ^a^ |  | Adjusted ^b^ |
|  |  | OR  (95% CI) |  | OR  (95% CI) |  | OR  (95% CI) |  | OR  (95% CI) |
| Duration of hearing-aid use |  |  |  |  |  |  |  |  |
| ≥ 8 to <12 hours (n=80) |  | 1.11 (0.45-2.49) |  | Ref |  | 0.47* (0.25-0.83) |  | Ref |
| < 8 hours (n=94) |  | 0.87 (0.35-1.95) |  | 1.79 (0.38-10.95) |  | 2.06* (1.26-3.38) |  | 3.63* (1.44-10.08) |
| ≥ 12 hours (n=158) |  | 1.04 (0.49-2.18) |  | 0.77 (0.29-2.13) |  | 0.90 (0.57-1.43) |  | 1.62 (0.82-3.28) |

^a^ Unadjusted estimates are derived from univariate logistic regression analyses.

^b^ Multivariate logistic regression analysis was performed adjusted for age, sex, education level, economic status, household, employment, aided ear,　history of ear disease, hearing-aid use method, frequency of hearing-aid use, period of hearing-aid use, pure-tone average (PTA), and fitting evaluation based on sound field.

p < .05
